# Pseudomyxoma Peritonei: A Case Report Diagnosed in a 47-Year-Old Woman with Chronic Pelvic Abdominal Pain and Appendicular Origin: Review of the Literature and Management

**DOI:** 10.3389/fsurg.2017.00041

**Published:** 2017-12-18

**Authors:** Francois Pugin, Jean Bouquet De Jolinière, Attila Major, Fathi Khomsi, Louis Guillou, Mathias Peter, Nordine Ben Ali, Bernhard Egger, Anis Feki

**Affiliations:** ^1^Department of Digestive Surgery, HFR, Cantonal Hospital of Fribourg, Fribourg, Switzerland; ^2^Department of Gynecology and Obstetrics, HFR, Cantonal Hospital of Fribourg, Fribourg, Switzerland; ^3^Department of Pathology, Argotlab, Lausanne, Switzerland

**Keywords:** pseudomyxoma peritonei, appendicitis, ovarian cancer, mucoid ovarian cyst, intraperitoneal chemotherapy, laparoscopic surgery

## Abstract

The authors report a case of pseudomyxoma peritonei with gelatinous peritoneum in a 47-year-old-woman. The main symptom for discovery was a chronic pelvic abdominal pain. This disease is particularly rare. The gelatinous substance is often associated with a malignant ovarian tumor or appendicitis perforated. Currently, on the whole, an exploratory laparoscopy allows diagnosis, biopsies, and appendectomy. The treatment is essentially surgical. The prognosis depends on grade (1/3) and response to chemotherapy. This case was presented to the tumor board.

## Observation

A 47-year-old woman without comorbidities presented to a gynecologist for a chronic pelvic abdominal pain for 4 months. There were no other signs. The patient is not at a menopausal stage and had the last period date before a week of admission. Also she is homosexual. She agreed to undergo eventual hysterectomy. Thoracoabdominal contrast-enhanced MRI and CT scan were then performed after an endovaginal ultrasound showing a left adnexal ovarian mass that is heterogeneous and little mobile.

The MRI shows a fluid mass of 10 × 7 × 8 cm encompassing the uterus and ovaries. It presents a fine peripheral enhancement onto which enhanced tissular nodules are grafted, and there are also septa behind the left ovary and a curvilinear calcification.

T2 sequences confirm a dilated appendix at 11 mm of diameter with thickened walls, containing liquid, and hypo signal T2, calcifications and especially an enhancement on the appendicular tail.

This examination shows a peritoneal pseudomyxoma with a partitioned mass encompassing the uterus and adnexa, nodular vesicular and tissue implants, and small mucoid implants scattered in the peritoneal cavity.

An appendicular mucocele and a gelatinous disease of the peritoneum are possible complications.

Radiologists requested an abdominal scan to visualize an ovarian malignant pathology. The results of this review are in contradiction with the MRI. The differential diagnosis revealed a stage III c ovarian cancer.

An exploratory laparoscopy is performed showing a gelatinous disease of the peritoneum. Both ovaries are ovulatory, with a normal wall. The appendix is retro cecal and dilated at its base with an infiltrated meso. Appendectomy is performed systematically. Its base is particularly dilated. A double suture by Endoloop is necessary.

The peritoneal lavage is then carried out. Mucus is sent to anatomical pathology department for the evaluation of the histological grade. Ovarian and peritoneal biopsies are performed. All results will be presented to the tumor board to decide on further treatment. No other abnormalities are found in the abdominal cavity.

## Review of the Literature

First, a gelatinous disease of the peritoneum indicates the presence of a gelatinous ascites, due to mucin-producing tumor cells implanted on the peritoneal surfaces. Mucin is essential for the diagnosis.

### History

In 1842, first Rokitansky and then Cruveilhier described gelatinous degeneration in the peritoneum and believed that the disease originated in the ovaries (1).

In 1871, Pean admitted the ovarian origin and the myxomatous nature of the disease and qualified it as “gelatinous disease of the peritoneum” (1).

Werth on 1884 described a gelatinous subperitoneal cavity due to the rupture of a pseudo mucous ovarian cyst with a gelatinous material but without mucine (1).

Frankel on 1901 described for the first time a rupture of appendicular cyst (1).

### Etiology

The main causes are the appendicular origin (mucocele appendicular; 30%), ovarian (mucinous carcinoma; 60%), and peritoneal causes. Other causes are described in the literature (1): malignant schwannoma and other peritoneal diseases from bowel. This disease is particularly rare (2 cases/1 million/year and 2 cases/10,000 laparotomies) (2) and can affect both sexes, especially females at the age of 50s (2).

The mucocele is due to the chronic nature of luminal distension. Most frequently, a rupture of a mucinous tumor or a mucocele of the appendix in the peritoneal cavity leads to the gelatinous disease of the peritoneum (2). This is the most severe complication with diffusion of the gelatin responsible for occlusion, severe adherences between abdominal organs (3, 4).

Dissemination initially is locoregional. But, it is frequent to see multiple sites of localization. Fairise et al. (5) described multiple zones of implantation knowing these cells have a low-adhesive potential. The privileged zones are those where stasis is possible (Douglas poach, parabolic gutters), but all abdominal organs are concerned (duodenal junction, antropyloric zone, right diaphragmatic dome, retro sigmoid region). But, the main problem is the posttraumatic and post-surgical scarring, explaining imperatively a surgical and chemotherapeutic management at the same time.

For a long time, it was believed that the origin could be mixed (ovarian and appendicular). Moreover, there are often voluminous ovarian tumors in this disease, which are mistaken for primitives. Immunohistochemistry and genetics history were to be expected to affect the appendicular mucocele as the main cause of the disease. Appendix is distended by hypersecretion of mucin: it is the mucocele appendicular (2).

Actually, research seems to show that the disease progression is related to microbial agents (MUC2 and MUC5AC expression in disseminated peritoneal adenomucinosis and peritoneal mucinous carcinomatosis) (6, 7). Multiple enteric bacteria are probably present in PMP (6). Ronnett et al. (5) have described two groups based on histopathological criteria used to characterize the most favorable prognosis for each of them.

If the appendix is the most frequent origin of the disease, other tumors may be concerned for the etiology: mature teratoma, digestive tumors, etc.

The three reasons for appendicitis are obstruction, distension, and rupture with intraperitoneal dissemination of the mucus. Each implant is an epithelial cell proliferation producing mucus (5). Dissemination can be to the whole abdominal cavity. The circulation of the fluid seems to affect mainly all the areas covered by the parietal peritoneum. The traumatized peritoneal zones seem to favor the implantation of the mucus. The great omentum is very often concerned by omental implants. It is the reason why the surgical treatment can be aggressive.

### Diagnostic

There are no specific signs. The main sign is abdominal ascites: from a simple effusion to an important ascites, in fact, the clinical aspect is rich (as transit disorders, digestive signs, signs of subocclusion).

But the absence of pathognomonic signs explains the difficulty of clinical diagnosis. In our case, only the pelvic abdominal pain was present. In published literature, it is always imagery and laparoscopic findings that offer the macroscopic diagnosis.

Biology is not useful for diagnosis, but tumor markers (CEA, CA 19.9) are used for the detection of early recurrences. So we need of a starting rate. They are high in most of the patients and useful to survey the chemotherapy efficiency.

Imaging (endovaginal ultrasounds, RMI, and TDM) are very useful to see the lesions.

Ultrasounds allow seeing an eventual ovarian cystic mass associated with peritoneal implants or effusion, evaluate septa, and calcifications.

They allow seeing the implants, the associated tumors (ovaries), the effusion, the extension of the disease on mesentery, and in deciding the surgical strategy.

They will show the “scalloping of the liver” (2), the partitions, the state of the omentum, and the peripheral enhancement after injection.

But, laparoscopy is the best indication to explore the peritoneal cavity. During the procedure, it is possible to evacuate the mucus and wash the cavity, to perform the biopsies of the peritoneum, and to carry out a diagnostic adnexectomy, a systematic appendectomy.

The aim is to obtain an anatomopathological analysis, to evaluate the appendix, and to define the grade and the stage of the disease because the therapeutics depends on it and to define a therapeutic strategy.

**Figure 1:**
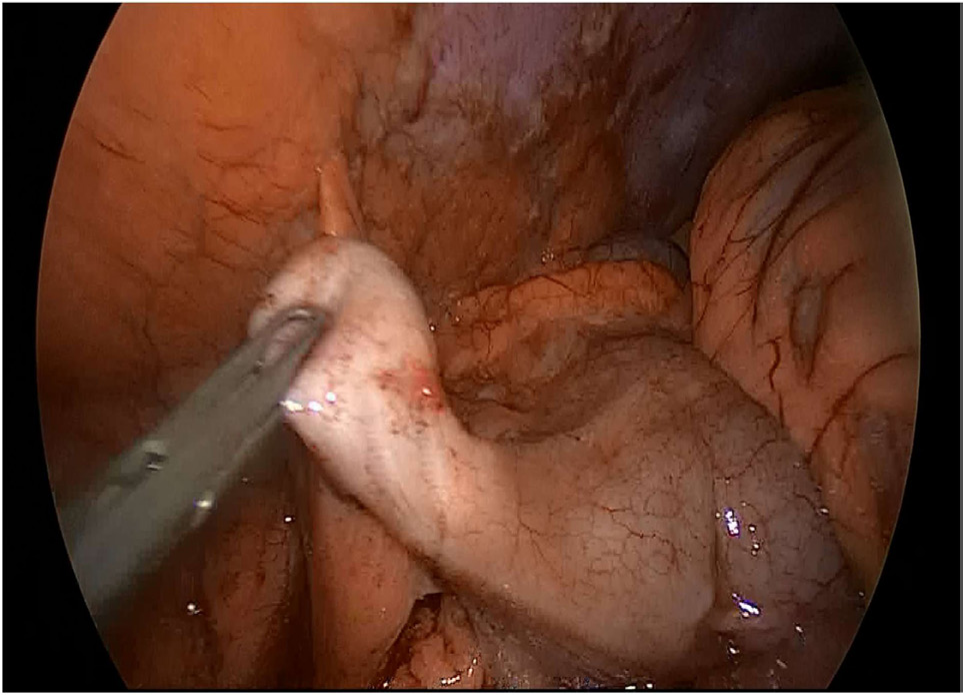
Appendicular pseudomyxoma with enlarged base.

**Figure 2:**
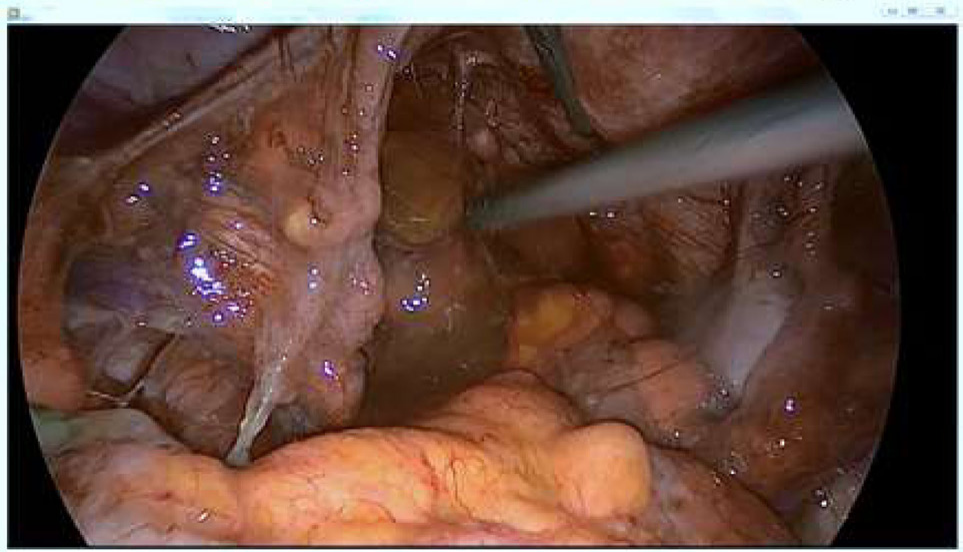
Gelatinous material in the Douglas’ pouch.

**Figure 3:**
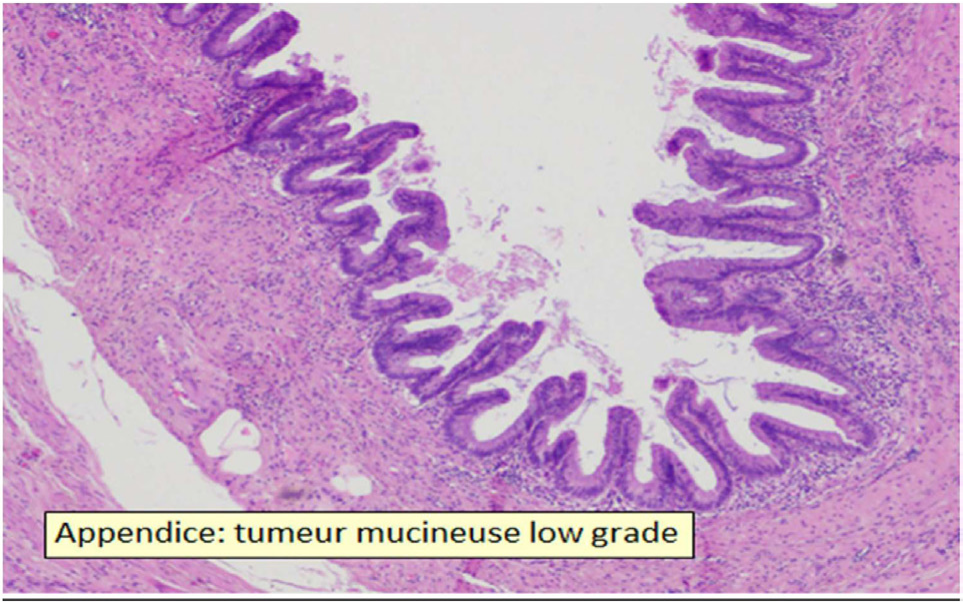
Appendix with low-grade mucinous tumor.

In the case of our patient, washing and aspiration were performed to remove the maximum of the gelatinous effusion, and staging was completed by performing biopsies of the peritoneum and the macroscopically normal ovaries. On the other hand, the appendix appeared abnormal, and we confirmed an appendectomy. (Its base showed a mucocele of 1.1 mm diameter.) The mesentery was normal and there were no lesions on the liver and stomach and omentum was normal macroscopically. The diaphragmatic domes are not yet reached (Figures 1–3).

### Results of the Patient’s Pathology

- Appendix, appendectomy: low-grade mucinous tumor limited to the mucosa extending over 2.4 cm in length, without submucosal infiltration (LAMN), without vascular invasion, complete excision, healthy section of the appendix without tumor, and small defect of the wall near the sectional section without associated inflammatory reaction.
- Douglas, biopsies: low-grade appendicular mucinous tumor (LAMN).
- Peritoneum: deposit of mucus without visible tumor epithelial cells.
- Peritoneal washing: rare atypical cells compatible with mucinous adenocarcinoma.

TNM classification for mucinous peritoneal tumor is as follows: pT4a, pNx, L0, V0, pPnO, and G1.

In Figure 1, the appendix is dilated to its base with no visible burglary or fistula. In Figure 2, the gelatinous effusion is visible in the cul-de-sac of Douglas, on the peritoneum of the right parietal colic gutter.

The ovaries show no macroscopic abnormalities but are covered with mucus. After washing, a bilateral biopsy is carried out for diagnostic purposes.

Exploratory laparoscopy was used for diagnosis and to perform peritoneal staging, appendectomy, and ovarian biopsies.

The final pathological result allows to determine the TNM classification and the stage of the disease.

The different sections show that there is no invasion of the appendix but the presence of adenocarcinomatous cells in the effusion (Figure 3).

TNM classification for mucinous peritoneal tumor: pT4a, pNx, L0, V0, pPnO, and G1.

### Details and Explanations of Pathology: WHO and TNM

The general classification into four grades has been previously described (Figure 3).

#### Group 1: Mucinous Cystadenomas

It is a mucinous neoplasia of low grade, with or without cysts, characterized by a proliferation of cylindrical epithelial cells, planar or villous architecture without mucus, neoplastic cells, and invasive extraappendicular focus.

#### Group 2: Mucinous Lesions with a Low Risk of Recurrence

It is an appendicular lesion of mucinous neoplasia of low grade, with or without cysts, characterized by a proliferation of cylindrical epithelial cells, flat or villous architecture with the presence of extraappendicular mucus, absence of neoplastic cells, and invasive extra-appendicular focus.

#### Group 3: Mucinous Lesions with a High Risk of Recurrence

Group 3 consists of lesions of mucinous neoplasia of low grade, with or without cysts, characterized by a proliferation of cylindrical epithelial cells, flat or villous architecture with the presence of mucus, extraappendicular neoplastic cells, and absence of invasive extraappendicular focus.

#### Group 4: Mucinous Adenocarcinoma

Group 4 consists of appendicular lesions of high grade, mucinous neoplasia, or invasive neoplasia invading the wall—appendicular beyond the mucous muscle.

There is possible presence of mucus, neoplastic cells, and extraappendicular invasive foci.

The results of the patient are as follows:

- Appendectomy: The material examined in total and over several depths shows multiple depressions of mucus and epithelial flaps bordered by a layer of mucus-secreting cylindrocellular epithelial cells with mild cytotoxic atypia. No high-grade dysplasia component is seen. The submucosa is thinned and atrophic. Epithelium in low-grade dysplasia is limited in the appendix, 0.3 cm from the surgical section and 0.8 cm from the tip. The tip is obstructed by a fibrous reshaping. At 0.3 cm from the surgical section, there is an area of defect of the appendicular wall, with no associated inflammatory reaction.
- About Douglas and peritoneum: fragments of low-grade appendicular mucinous tumor and fragments of peritoneum in the presence of mucus deposits mixed with chronic and acute inflammatory elements, without tumor epithelial cells.

The peritoneal lavage shows rare atypical cells compatible with a mucinous adenocarcinoma ++.

WHO histological classification of the tumors of appendix.

**Table:** 

Epithelial tumors	Non-epithelial tumors
Adenoma: tubular, villous, tubulovillous, serrated	Neuroma
	Lipoma
	Leiomyoma

Carcinoma: adenocarcinoma, mucinous adenocarcinoma, signet-ring cell carcinoma, small cell carcinoma, undifferentiated carcinoma	Gastrointestinal stromal tumor
	Leiomyosarcoma
	Kaposi sarcoma

Carcinoid: well-differentiated endocrine neoplasm: EC-cell, serotonin-producing neoplasm, L-cell, glucagon-like peptide, PP/PYY-producing tumor, others	

Tubular carcinoid	Malignant lymphoma

Goblet cell carcinoid (mucinous carcinoid)	Secondary tumors

Mixed carcinoid—adenocarcinoma	Hyperplastic (metaplastic polyp)
Others

### Imaging

Abdominal radiographs are rarely helpful for diagnosis (5). Ultrasound, MRI, and CT scan are the preferred methods to evaluate effusions, tissular lesions, and ovaries.

#### Commentary about Patient’s RMI

The MRI shows a fluid mass of 10 × 7 × 8 cm encompassing the uterus and ovaries. It presents a fine peripheral enhancement onto which enhanced tissular nodules are grafted, and there are also septa behind the left ovary and a curvilinear calcification.

The masses do not show a hyper signal in diffusion and there is no decrease in the diffusion coefficient.

A complementary ultrasound is performed, which shows heterogeneous fluid mass that do not move around the left ovary, without mobilization to the right during the right lateral decubitus.

**Figure 4:**
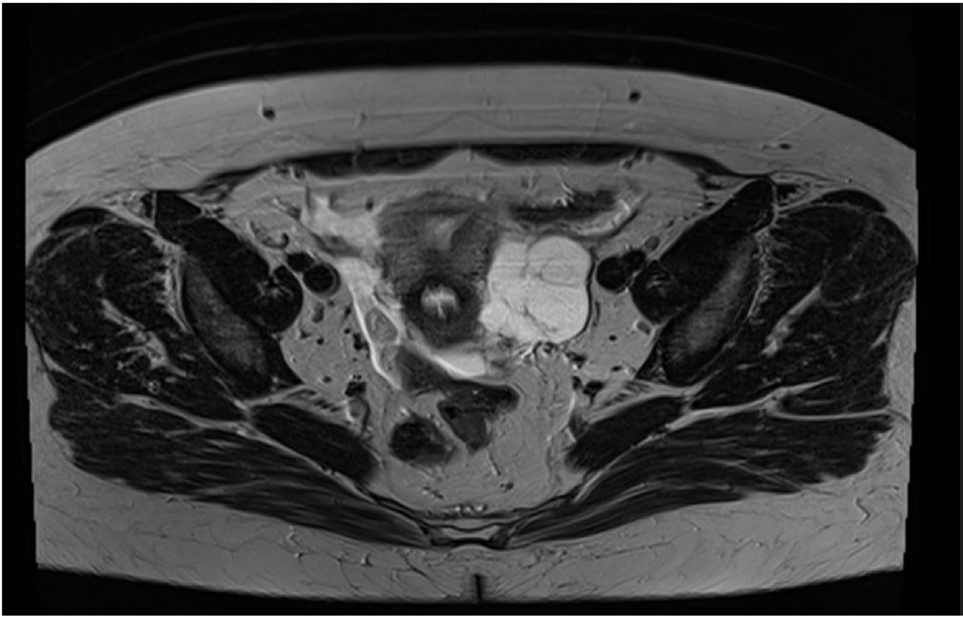
Cross-section of RMI with effusion around the uterus.

**Figure 5:**
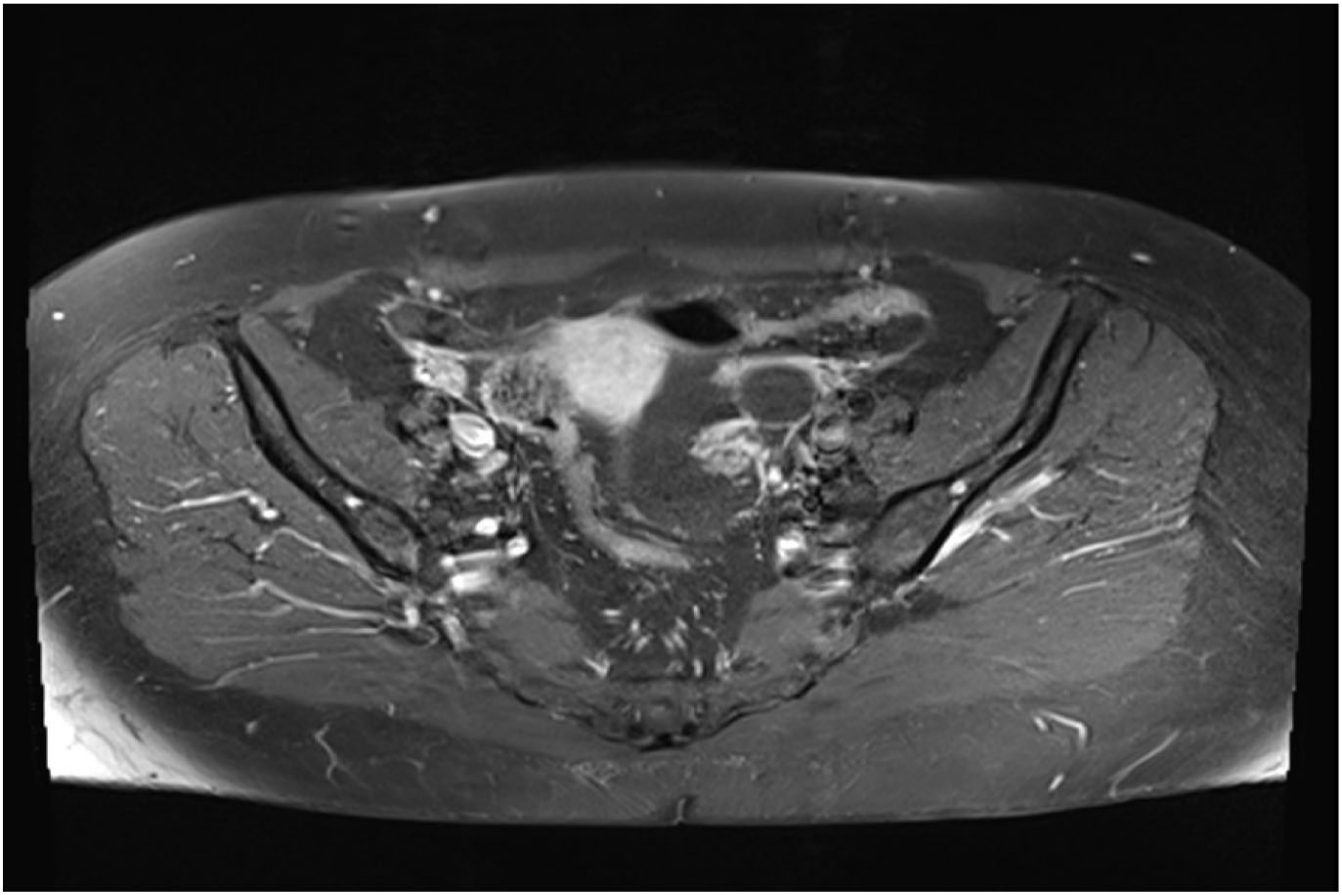
CT view of abdominal scan showing that the left ovary is independent of the effusion.

The coronal sequence in abdominal T2 shows small nodular fluid implants scattered in the peritoneum under the mesocolic zone, at the tip of the right liver (no visible lesions) without “scalloping” (Figures 4 and 5).

But this sequence shows a cecal appendix dilated to 11 mm in diameter and 37 mm in length, containing liquid with thickened walls, in hyposignal T2 with calcifications, and an elevation in relation to the appendicular tail with tissue thickening.

The MRI is compatible with peritoneal pseudomyxoma, but appendicular mucocele and gelatinous disease of the peritoneum remain possible (Figures 1, 3–5).

It is a probable appendicular mucocele at the origin of this pseudomyxoma.

### Discussion about the Recommendations Found in the Literature Regarding Treatment

The mainstay of the treatment is surgery and chemotherapy. Radiotherapy is not efficient because the tumor is not differentiated.

In our case, washing and aspiration removed the maximum of the mucous effusion, and laparoscopy allowed us to visualize normal ovaries, but a bilateral biopsy was performed, and the systematic appendectomy (diameter of the base of 1.1 cm) must allow the diagnosis of pseudomyxoma, define the grade, and decide on the surgical and chemotherapeutic strategy. Surgery must remove all lesions to eradicate the lesional process. In case of malignant mesothelium, all peritoneal lesions should be removed: but the surgical time and the postoperative complications make this surgery extremely difficult (2).

A good washing of the peritoneal cavity is useful, but the recurrence is very frequent. The approach depends on the size of the lesions and damage. But laparotomy remains a good method. The surgery must obligatorily remove the appendix, sometimes even a right hemicolectomy and hysterectomy with bilateral adnexectomy.

In fact, intraoperative chemotherapy (8–11) and postoperative chemotherapy significantly improve the prognosis. This kind of treatment remains difficult and needs a specialized center. As in ovarian cancer, the effect of systemic chemotherapy remains limited on the peritoneum due to low-tissue penetration (8). This is why some authors propose pressurized intraperitoneal aerosol chemotherapy. This is apparently more effective in avoiding the frequent complications of the hyperthermic form (CHIP) (8, 11–13). But this treatment is still experimental and can be carried out only in specialized centers.

Taking advantage of the barrier between plasma and peritoneum, higher concentrations of chemotherapeutics penetrate peritoneal nodules with a low rate of systemic absorption. It is a real minimal invasive chemotherapy and repetitive (one application per month for 3 months). The procedure is performed for 90 min under general anesthesia. The chemotherapy is vaporized in the form of an aerosol, which is therefore homogeneous in the peritoneal cavity benefiting from laparoscopy under a pressure of 12 mm Hg (8). In literature, the postoperative morbidity is less than 20%. The laparoscopy allows the exploration of peritoneal cavity and defines a peritoneal carcinoma index. Coupled with repeated biopsies before and after treatment, it allows the objective evaluation of response to treatment (8).

The problem of peritoneal disease is that it remains microscopic, and surgical redux would require removal of the entire peritoneum, but this long and dangerous surgery can result in large complications or even vital prognosis. It will remain incomplete since it is not possible to remove all mesos. Moreover, no current imaging can visualize peritoneal lesions in preoperative and postoperative surgery. Tumor blood markers are ineffective and do not evaluate response to treatment.

Finally, in three studies, the prognosis depends on complete cytoreductive surgery and association with hyperthermic intraperitoneal chemotherapy. Surgery should be continued if possible. The recurrence rate remains high, and preoperative and postoperative morbidity remains high at 24%. Mortality is estimated at 2% (14–17).

## Author Contributions

All authors participated in this article.

## Conflict of Interest Statement

The authors declare no conflict of interest and not any ethical problems. A written informed consent was obtained from the patient for the publication of this case report. The reviewer, BH, and handling editor declared their shared affiliation, and the handling editor states that the process nevertheless met the standards of a fair and objective review.
